# HMGB1 Inhibition to Ameliorate Organ Failure and Increase Survival in Trauma

**DOI:** 10.3390/biom12010101

**Published:** 2022-01-08

**Authors:** Zhangsheng Yang, Milomir O. Simovic, Peter R. Edsall, Bin Liu, Tomas S. Cancio, Andriy I. Batchinsky, Leopoldo C. Cancio, Yansong Li

**Affiliations:** 1 US Army Institute of Surgical Research, JBSA Fort Sam Houston, San Antonio, TX 78234, USA; Zhangsheng.yang.ctr@mail.mil (Z.Y.); msimovic@genevausa.org (M.O.S.); peter.r.edsall.civ@mail.mil (P.R.E.); bin.liu4.ctr@mail.mil (B.L.); tomas.s.cancio.ctr@mail.mil (T.S.C.); abatchinsky@genevausa.org (A.I.B.); 2 The Geneva Foundation, Tacoma, WA 98402, USA

**Keywords:** HMGB1, trauma, hemorrhagic shock, inflammation, multiple organ failure

## Abstract

Several preclinical and clinical reports have demonstrated that levels of circulating high mobility group box 1 protein (HMGB1) are increased early after trauma and are associated with systemic inflammation and clinical outcomes. However, the mechanisms of the interaction between HMGB1 and inflammatory mediators that lead to the development of remote organ damage after trauma remain obscure. HMGB1 and inflammatory mediators were analyzed in plasma from 54 combat casualties, collected on admission to a military hospital in Iraq, and at 8 and 24 h after admission. In total, 45 (83%) of these patients had traumatic brain injury (TBI). Nine healthy volunteers were enrolled as controls. HMGB1 plasma levels were significantly increased in the first 8 h after admission, and were found to be associated with systemic inflammatory responses, injury severity score, and presence of TBI. These data provided the rationale for designing experiments in rats subjected to blast injury and hemorrhage, to explore the effect of HMGB1 inhibition by CX-01 (2-*O*, 3-*O* desulfated heparin). Animals were cannulated, then recovered for 5–7 days before blast injury in a shock tube and volume-controlled hemorrhage. Blast injury and hemorrhage induced an early increase in HMGB1 plasma levels along with severe tissue damage and high mortality. CX-01 inhibited systemic HMGB1 activity, decreased local and systemic inflammatory responses, significantly reduced tissue and organ damage, and tended to increase survival. These data suggest that CX-01 has potential as an adjuvant treatment for traumatic hemorrhage.

## 1. Introduction

Trauma/hemorrhage (TH) is the leading cause of death on the battlefield, and, for those under the age of 45, in the civilian world as well [1]. However, two thirds of severe trauma patients succumb to causes other than exsanguination [2,3]. Tissue injury and hemorrhage involve ischemia and reperfusion injury (IRI) and initiate a series of prompt innate immune responses, including both pro-inflammatory and anti-inflammatory processes. In the event of severe injury, the inflammatory response to blood loss and tissue damage can start within minutes [3,4,5,6].

Trauma immediately induces release of damage-associated molecular patterns (DAMPs), such as high mobility group box 1 protein (HMGB1, also known as amphoterin) [7,8,9], IL-1, IL-33, calgranulins, histones, heat-shock proteins, nucleic acids, adenosine triphosphate (ATP) [9,10,11], matricryptins [12], free heme [9,13], cold-inducible RNA-binding protein [14], and mitochondrial DNA [15]. DAMPs in conjunction with pathogen-associated molecular patterns (PAMPs) promptly activate the complement [16], coagulation [17], and kallikrein-kinin systems, [18], ultimately leading to trauma-induced immune dysfunction, infectious complications, and multiple-organ failure (MOF). Although trauma care has gradually improved, therapeutic treatment of these sequelae remains challenging.

HMGB1, which belongs to the superfamily of high mobility group (HMG) proteins, is a highly conserved non-histone nuclear protein and ubiquitously expressed in almost all cells (i.e., monocytes, macrophages, etc.). HMGB1 contains two lysine-rich nuclear localization signal (NLS) domains located in A box and B box, respectively, and an acidic tail. Under physiological conditions HMGB1 is predominantly present inside nucleus through its NLS binding with nuclear cargo carrier proteins. HMGB1 shuttles from the nucleus to the cytoplasm via NLS acetylation, phosphorylation, or methylation, and can both passively (via damaged and/or necrotic cells) and actively (via activated monocytes/macrophages) release into extracellular space and circulation. The biological functions of HMGB1 are dependent on its location: (1) nuclear HMGB1 is involved in several nuclear processes, such as chromatin stabilization, replication, DNA-damage repair and gene transcription; (2) cytoplasmic HMGB1 participates in the regulation of AIM2 (absent in melanoma 2) inflammasome, autophagy, and mitophagy; and (3) extracellular HMGB1 (eHMGB1) functions as a proinflammatory mediator [19,20]. The translocation of HMGB1 between cellular compartments is a dynamic process triggered by variety of cell stresses and diseases (sepsis, trauma, hemorrhagic shock, ischemia, etc.) [19,20,21,22].

Extracellular HMGB1 has a strong pro-inflammatory effect [23]. Although eHMGB1 as a host product is sterile itself, it signals through toll-like receptors (TLR2, TLR4), the receptor for advanced glycation end products (RAGE), NF-κB-inflammasome, and/or CXCL-12-CXCR4-NF-κB-inflammasome axis, and induces organ damage even in the absence of infection [22,24,25,26]. HMGB1 released from damaged/necrotic tissue and the activated innate immune system [27,28] signals tissue damage to macrophages and dendritic cells (DCs) to secret HMGB1 in a vicious-circle pattern. Thus, eHMGB1 holds a central role in conveying the local and systemic responses to noxious factors, including trauma and sepsis [29].

Our previous studies have shown the beneficial effects of pharmacological manipulation of complement activity on survival, hemodynamics, fluid requirements, tissue damage, and local and systemic inflammation in rats and swine in short-term studies (<6 h) after TH [30,31,32,33]. In experiments with rats exposed to TH, we have recently shown that the inhibition of the complement terminal pathway (CTP) reduced eHMGB1 levels in the blood, and coincided with beneficial treatment (unpublished data). This finding, and the upstream position of eHMGB1 relative to the plasma cascade systems [16,17,18], identify eHMGB1 as a biomarker and therapeutic target in processes in which inflammation plays a key role [34,35].

Heparin and heparin-derived molecules demonstrated their anti-HMGB1 properties via direct heparin-HMGB1 binding and/or indirect blockade of RAGE/TLR2/4-HMGB1 interaction [36,37,38]. Although both unfractionated heparin and low-molecular-weight heparin have anti-HMGB1 and anti-inflammatory properties, their anticoagulant activity limits their use in TH. CX-01 (2-*O*, 3*-O*-desulfated heparin) is a novel HMGB1 inhibitor with <5% anticoagulant activity [39,40,41]. We hypothesized that treatment with CX-01 would alleviate tissue damage and improve survival in a rat model of TH that recapitulates the immunological responses seen in injured patients [42].

## 2. Materials and Methods

### 2.1. Clinical Study Design

The Institutional Review Board (IRB) of Brooke Army Medical Center reviewed and approved the research protocol and granted a waiver of consent for blood sampling as a minimal-risk intervention. The study in trauma patients was designed to identify the clinical significance of early HMGB1 release in military casualties admitted to a US Army Combat Support Hospital (Role 3) in Baghdad, Iraq over a one-year period. Foreign nationals, prisoners, enemy combatants, children, and any patient undergoing therapeutic anticoagulation were excluded. Citrated plasma was collected from trauma patients after admission to the emergency department (n = 54, 53 males and 1 female; median age =25 years, interquartile range from 22 to 30 years), and, if available, 8 h (n = 23) and 24 h (n = 9) later. At these later time points, samples were collected after patients had received appropriate clinical care, including surgery and resuscitation. On admission (45–60 min after injury) and during the first 24 h, the clinical characteristics and demographic information of the patients were recorded, including base excess, mean arterial pressure (MAP), blood product transfusion units, and systemic inflammatory response syndrome (SIRS) score. Most casualties (83%, n = 45) suffered traumatic brain injury from explosions. Of the 9 healthy controls, 5 were male and four were female with a mean age of 35.4 ± 9.1 years.

Blood collected at the hospital was processed according to standard clinical practice. Briefly, blood was drawn into citrated tubes and centrifuged for 15 min at 4 °C immediately after collection. Plasma was removed immediately after centrifugation and frozen and transported to the US Army Institute of Surgical Research as described in [43] and stored at −80 °C until analysis. Nine healthy volunteers were enrolled at the authors’ laboratory as reference controls. Volunteers were 18 years or older with no significant medical conditions. Blood samples were collected once for the analysis of the levels of HMGB1, cytokines, selected complement components and blood chemistry.

### 2.2. Animal Study Design

The USAISR Institutional Animal Care and Use Committee approved all research conducted in this study. Experiments in rats subjected to TH were used to evaluate the efficacy of drug CX-01 (Chimerix, Inc., Durham, NC, USA). The effects of inhibition of HMGB1 on tissue damage and survival of the injured rats were examined to verify the therapeutic potential of CX-01.

After recovery (5–7 days) from surgical cannulation and prior to TH, animals were randomly assigned to one of three groups: (1) the injured/untreated group (n = 9) underwent blast injury and hemorrhage (B + H), and sham treatment with saline vehicle; (2) the injured/treated group (CX-01, n = 6) underwent blast injury and hemorrhage, and treatment with CX-01; and (3) the time-control group (Control, n = 6) underwent all procedures except B + H and subsequent sham treatment. There were no exclusions from the experiments, and drug and vehicle administration were non-blinded. A randomized blinded code for histological sections was used.

### 2.3. Surgical Procedures and Trauma Induction in Rats

**Figure 1:**
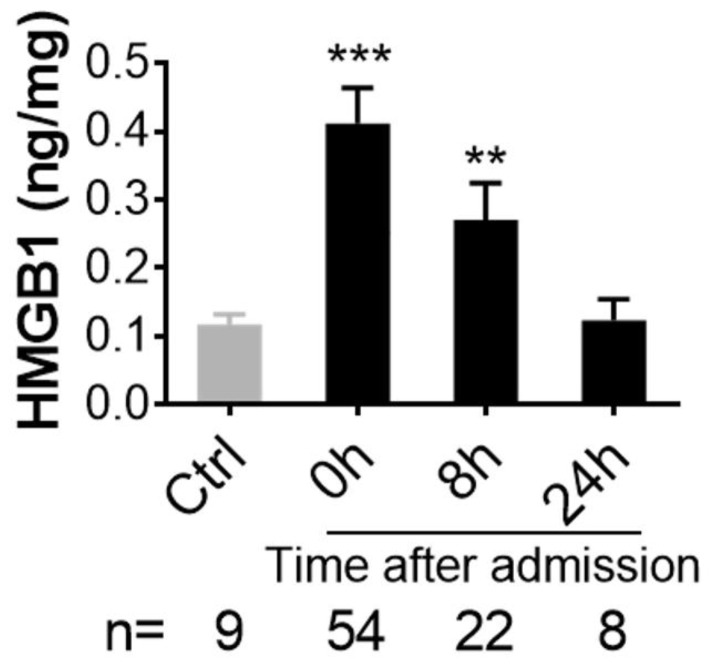
HMGB1 is significantly elevated after injury in trauma patients. Blood plasma from 54 casualties (on admission 8 and 24 h after admission to a hospital) and 9 civilian volunteers (Control, “Healthy”) was used for analysis. High mobility group box protein 1 (HMGB1), an inflammatory factor, was measured by ELISA. The data are expressed as nanograms per milligram total plasma proteins and presented as mean ± SEM, ** = *p* < 0.01, *** = *p* < 0.001 vs. Control (Ctrl).

Specific pathogen-free adult male Sprague-Dawley rats (10–12 weeks old), weighing 350–475 g were purchased from Charles River Laboratories (Wilmington, MA, USA) for use in this study. Under anesthesia of 1–2.5% isoflurane, the carotid artery and jugular vein were cannulated in all rats. The cannulated animals went through a 5–7 day recovery period. Blast injury was conducted as described previously [44,45] (Figure 1A). Briefly, the rats were anesthetized with ketamine/xylazine (60/5 mg/kg body weight) via intra-peritoneal injection, and then placed on a rack holder, which was wheeled into the end of the expansion chamber of a compressed air-driven shock tube [42]. During the blast process, the animals were immobilized to prevent movement by blast impact and subsequent secondary or tertiary blast injuries. The animals in the prone position with their heads turned to the blast wave were exposed to single mild-moderate blast injury. Blast severity was similar for both groups. For the sham group, blast data were BOP = 117.26 ± 1.36 kPa, t+ = 3.29 ± 0.01 ms, and I = 141 ± 0.87 kPa-ms. For the CX-01 group, blast data were BOP = 117.48 ± 1.82 kPa, t+ = 3.38 ± 0.03 ms, and I = 145 ± 0.39 kPa-ms (Table 1). Then, 15 min after blast exposure, animals were subjected to volume-controlled hemorrhage over 15 min. The 52% estimated total blood volume (ETBV) was calculated using the following formula: ETBV (mL) = weight in kg × 60 mL/kg. After hemorrhage, the animals were maintained for 30 min (shock phase), then received two times the shed blood volume of Plasma-Lyte A. The animals were monitored under anesthesia for 3 h after the end of the shock phase (H), then returned to their cages and observed for 24 h. In the CX-01 group, three doses of CX-01 (25 mg/kg of body weight) were administered. The first dose was given intravenously 15 min after blast but before hemorrhage, the second dose was administered at 2 h after blast, and the third dose was administered subcutaneously at 10 h after blast. The CX-01 dose of 25 mg/kg reflects the standard human unfractionated heparin intravenous dosage and has previously shown to result in a near complete inhibition of airway HMGB1 release in mice with *Pseudomonas* pneumonia [41]. Rats in the sham group received equal volumes of saline at the same time points.

During the observation period, the mean arterial pressure was recorded by a BIOPAC data acquisition system (BIOPAC Systems, Inc., Goleta, CA, USA). Blood samples were collected before the blast, 15 min after blast, at the end of hemorrhagic shock (60 min after blast), and at 2, 4, and 25 h blast after injury. The blood chemistry was analyzed using an i-STAT (Abbott Laboratories, Chicago, IL, USA), and the PaO_2_/FiO_2_ ratio (PFR) was based on collected i-STAT data.

### 2.4. Assays

#### 2.4.1. Assessment of Complement Factors in Human/Rat Plasma

Quantitative levels of complement factors in human plasma, including C3a, C5a, sC5b-9, Bb, and C4d, were measured using commercial enzyme-linked immunosorbent assay (ELISA) kits according to the manufacturer’s instructions (Quidel, San Diego, CA, USA). Rat plasma levels of complement C3 and C1q were assessed using ELISA kits (abcam, Cambridge, MA, USA).

#### 2.4.2. Measurement of Human Cytokines

Human cytokines in plasma were analyzed using Bio-Plex^®^ Pro Human Cytokine 27-plex Assay (BIO-RAD, Hercules, CA, USA) according to the manufacturer’s instructions.

#### 2.4.3. Analysis of Total Protein in Human Plasma

Levels of total protein in plasma were measured using a bicinchoninic acid protein assay kit (Pierce, Rockford, IL, USA) according to the manufacturer’s instructions.

#### 2.4.4. Hemolytic Complement Activity Assay

The complement hemolytic 50% activity (CH50) assay was performed to determine the function of the complement classical pathway, as previously described [45]. Briefly, antibody-sensitized chicken red blood cells (Colorado Serum Company, Denver, CO, USA, catalog #31151) were incubated for 30 min at 37 °C with serial dilutions of rat serum samples in gelatin-veronal buffer (GVB^++^ buffer, Complement Technology, Tyler, TX, USA, catalog #B100). After centrifugation, the supernatant was transferred to a new plate, and the absorbance of supernatant was determined at 405 nm by SpectraMax microplate reader (Molecular Devices San Jose, CA, USA). The fold serum dilution inducing 50% of complement hemolytic activity was determined and presented as the CH50 value.

#### 2.4.5. Measurement of HMGB1 and MPO Levels in Human and Rat Plasma

HMGB1 and myeloperoxidase (MPO) were measured using ELISA kits obtained from IBL International (Morrisville, NC, USA) and Hycult Biotech (Plymouth Meeting, PA, USA), respectively. HMGB1 and MPO were determined by quantitative sandwich enzyme-linked immunosorbent assay (ELISA) according to the manufacturer’s instructions.

### 2.5. Tissue Pathological Evaluation and Semi-Quantitative Scoring

Histological images for each individual rat tissue were recorded with 10× objective under a slide scanner (Axio Scan. Z1 v1.0, Zeiss, Germany), and representative images of each group were presented (magnification, 400×). Semi-quantitative scoring of lung, brain, and liver tissues was performed by a pathologist blinded to the treatment information, and the criteria for the evaluation of histological injury scores are as follows.

For the lung injury score, four parameters (alveolar fibrin edema, alveolar hemorrhage, septal thickening, and intra-alveolar inflammatory cells) were scored on each hematoxylin and eosin (H&E) stained slide based on: (1) severity (0: absent; 1, 2, 3, and 4 for increasingly severe changes); and (2) the extent of injury (0: absent; 1: <25%; 2: 25–50%; 3: 50–75%; 4: >75%). Total injury score for each slide was calculated as the sum of the severity plus the extent of injury [32,42]

For brain injury score, we undertook the approach previously described [42]. Two parts of the brain tissue were scored, including the frontal cortex and hippocampus. Damage was assessed using 5 distinct morphological parameters: neuronal morphological changes (shrinkage of the cell body, pyknosis of the nucleus, disappearance of the nucleolus, and loss of Nissl substance, with intense eosinophilia of the cytoplasm), neuronal loss, cytotoxic edema, vasogenic edema, and inflammatory cell infiltration in the brain cortex. The changes were scored according to their extent (score 0, 1, 2, 3, and 4 for an extent of 0%, <25%, 25–50%, 50–75%, and 75–100%, respectively) and the severity of the injury (score 0 = normal histology, score 1 = slight, 2 = mild, 3 = moderate, and 4 = severe alterations).

For the hepatic injury score, four parameters, including vascular congestion, hepatocyte death, degeneration, and inflammation were considered [32], and these parameters were assayed for severity (score 0 for no change, score 1, 2, 3, and 4 for more severe changes) and for the extent of injury (0: absent; 1: <25%; 2: 25–50%; 3: 50–75%; 4: >75%). The injury score represents the sum of the extent and the severity of injury.

### 2.6. Statistical Analysis

All statistical analyses were performed using GraphPad Prism 6.0 (GraphPad Software, San Diego, CA, USA). Demographic data are presented as interquartile ranges (IQR) or percentages as appropriate. Other data are presented as mean ± SEM. Intergroup comparisons for complement factors and cytokines in patients or animals were assessed by Mann–Whitney U test, or two-tailed unpaired *t-*test with Welch’s correction. Correlation analyses were analyzed by Spearman’s rank correlation test. For animal survival analysis, the log-rank Mantel–Cox test was performed. Two-way ANOVA was performed to compare the animal groups on particular variables (*p* < 0.05 was considered significant). All data were included and none were treated as outliers.

## 3. Results

### 3.1. Patient Demographics and Clinical Outcomes

We investigated 54 injured combat casualties, with injuries from blast exposure (68%, n = 37), gunshot wound (GSW, 26%, n = 14), burns (4%, n = 2), and one motor vehicle accident (2%, MVA). In total, 45 (83%) of the 54 patients had traumatic brain injury (TBI), of whom most (80%) sustained a mild TBI. During the hospital treatment, mechanical ventilation was used in 7 patients (13%), and 5 patients (9%) died. All patients received operative care and fluids.

### 3.2. HMGB1 Plasma Levels, and Correlations with Inflammatory Mediators and Clinical Features in Military Casualties

**Figure 2:**
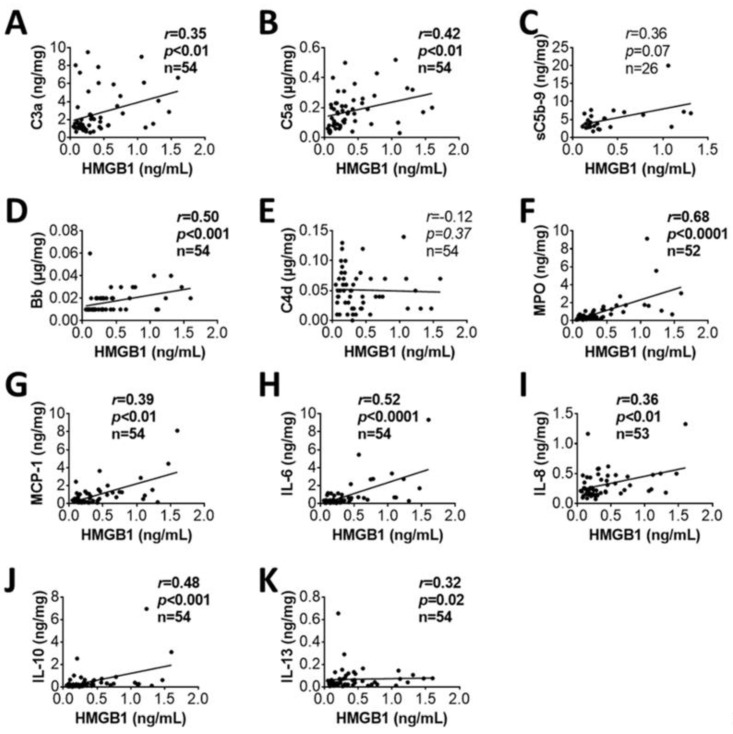
HMGB1 correlated with inflammatory mediators in trauma patients on admission. Correlations between plasma concentrations of HMGB1 in the casualties and C3a (**A**), C5a (**B**), sC5b-9 (**C**), Bb (**D**), C4d (**E**), MPO (**F**), MCP-1 (**G**), IL-6 (**H**), IL-8 (**I**), IL-10 (**J**), and IL-13 (**K**) in the blood plasma of the patients on admission are shown. Correlation analyses were performed by using Spearman’s rank correlation, and the data are presented with coefficient (r_s_) and *p*-values. Significant correlations (*p* < 0.05) are indicated by boldface type.

**Figure 3:**
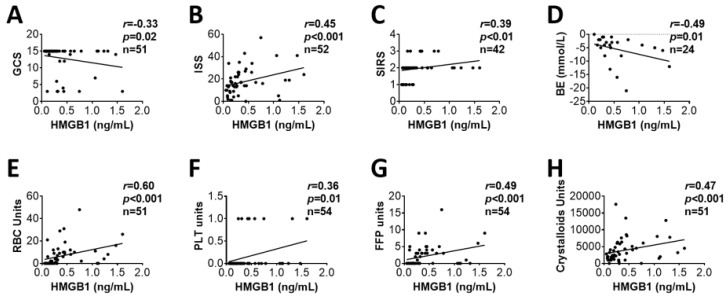
HMGB1 blood plasma concentrations correlated with clinical outcomes in trauma patients on admission. Positive correlations of HMGB1 blood plasma levels with ISS (**B**), SIRS (**C**), units of RBCs (**E**), units of platelets (**F**), units of fresh frozen plasma (**G**), and infused fluid (**H**) are shown. Negative correlations of HMGB1 plasma levels with GCS (**A**), and BE/BD (**D**) were observed. Correlation analyses were performed by using Spearman’s rank correlation, and the data are presented with coefficient (r_s_) and *p*-values. Significant correlations (*p* < 0.05) are indicated by boldface type.

Plasma levels of HMGB1 were significantly higher in combatants on admission to hospital and at 8 h after admission when compared to healthy controls (Figure 1). The HMGB1 plasma levels correlated positively with blood inflammatory mediators including activated complement factors (C3a, C5a, Bb, Figure 2A,B,D), MPO (Figure 2F), pro-inflammatory chemokine (MCP-1, Figure 2G), pro-inflammatory cytokines (IL-6, IL-8, Figure 2H,I), and anti-inflammatory cytokines (IL-10, IL-13 in Figure 2J,K, respectively) on admission. There was no correlation between HMGB1 and C4d (Figure 2E). The HMGB1 plasma levels on admission also correlated positively with ISS, SIRS, BE/BD (Figure 3B,C,D, respectively), transfusion units of RBCs, PLTs, FFP and infusion units of crystalloids (Figure 3E,F,G,H, respectively). The Glasgow Coma Scale (GCS), a tool used for the assessment of a patient’s consciousness, correlated negatively with HMGB1 plasma levels (Figure 3A).

### 3.3. CX-01 Partly Improved Hemodynamics but Not Blood Chemistries

**Figure 4:**
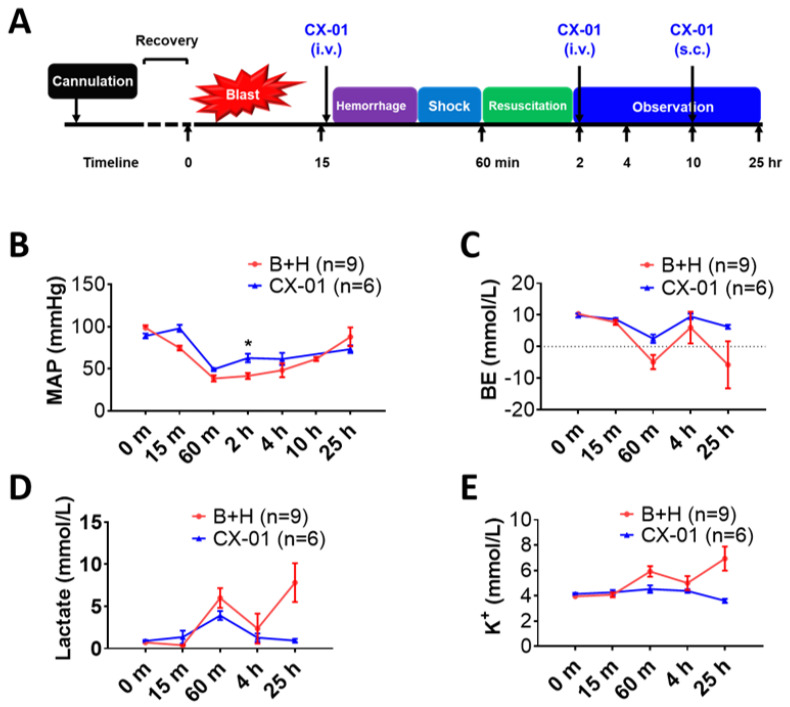
Effects of CX-01 treatment on MAP, and blood chemistry changes in the rat model of TH. (**A**) Experimental design. Experimental groups: (1) B + H = blast overpressure + hemorrhagic shock; (2) CX-01-treated injured rats. As marked in (**B**) changes of MAP were monitored via the carotid artery by the BIOPAC system and the changes were recorded throughout the study. During shock and resuscitation period, the MAP was recorded every 5 min; (**C**) BE/BD; (**D**) lactate; and (**E**) potassium plasma concentrations are given. Data are presented as mean ± SEM. The individual time points were compared using the unpaired *t*-test with Welch’s correction, and comparison of the groups was performed by two-way ANOVA. * = *p* < 0.05 Labels: MAP = mean arterial pressure; BE/BD = base excess/base deficit; K^+^ = ionized potassium.

The MAP in the injured animals was 98.5 ± 3.0 mmHg at baseline (Figure 4B) and decreased to 38.5 ± 3.7mmHg at the end of hemorrhagic shock (60 min). The MAP was significantly higher two hours post-blast (62.6 ± 5.0 mmHg) in the injured animals treated with CX-01, but showed no statistical difference when compared with the non-treated injured group (41.4 ± 3.7 mmHg). There was no statistically significant difference in the MAP between the two experimental groups in further observation. There was no clear difference in BE/BD (Figure 4C), blood lactate (Figure 4D), and potassium levels (Figure 4E) between two groups.

### 3.4. CX-01 Reduced Systemic Inflammatory Responses

**Figure 5:**
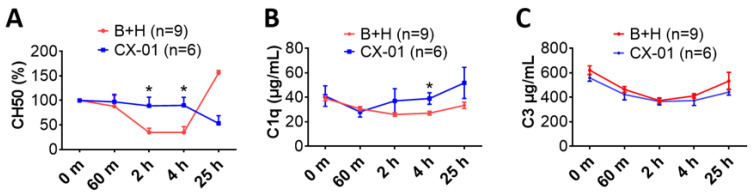
Effects of CX-01 treatment on complement hemolytic activity and complement consumption in the rat model of TH. (**A**) The hemolytic activity (CH50) of sera was measured, and the CH50 at each time point was normalized to the baseline level, which was pre-blast injury and the percentages of normalization are shown; (**B**) C1q blood serum concentrations; and (**C**) C3 blood serum concentrations. Data are presented as mean ± SEM. The individual time points were compared using the unpaired *t*-test with Welch’s correction, and comparison of the groups was performed by two-way ANOVA. * = *p* < 0.05 Labels: B + H = blast + hemorrhagic shock; CX-01—treated injured rats (more details in Figure 4A).

**Figure 6:**
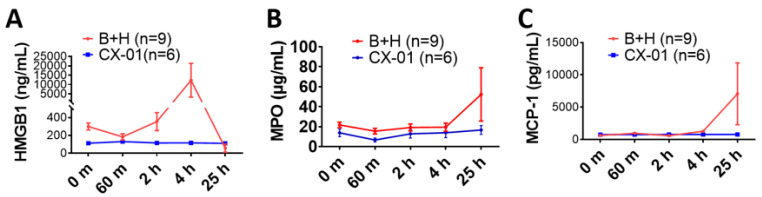
Effects of CX-01 treatment on cytokine concentrations in the rat model of TH. (**A**) HMGB1 plasma concentrations; (**B**) MPO plasma concentrations; and (**C**) MCP-1 plasma concentrations. Data are presented as mean ±SEM. Data were compared by using unpaired *t*-test with Welch’s correction, and comparison of the groups was performed two-way ANOVA. Labels: B + H = blast + hemorrhagic shock; CX-01—treated injured rats (more details in Figure 4A).

The hemolytic activity (CH50) in the blood of the untreated injured rats (group B + H) started to decrease at the end of hemorrhagic shock (60 min), reaching the lowest level at 2 h, was maintained for the following two hours, but by the end of observation period the CH50 exceeded the baseline level (Figure 5A). In the CX-01-treated injured rats, the CH50 at 2- and 4-h post-blast was significantly higher than the CH50 value in the non-treated group. This showed that the CX-01 reduced complement consumption. The consumption of C1q was also reduced in the CX-01-treated injured rats at 4 h when compared to the animals not treated with the drug (Figure 5B), but there was no clear difference in the consumption of C3 between the two experimental groups (Figure 5C). The HMGB1 blood levels in the rats not treated with CX-01 (B + H group) were obviously elevated at 2 h, peaked at 4 h, and returned to baseline levels at 25 h post-blast injury. Early treatment with CX-01 suppressed plasma HMGB1 activity but did not reach a statistically significant difference (Figure 6A). The HMGB1 blood concentrations appeared to remain at the basal level in the drug-treated injured animals. There was no clear difference in the MPO (Figure 6B) and MCP-1 (Figure 6C) blood levels between the drug-treated injured rats and those non-treated.

### 3.5. Effects of CX-01 on Multiple-Organ Damage and Survival

**Figure 7:**
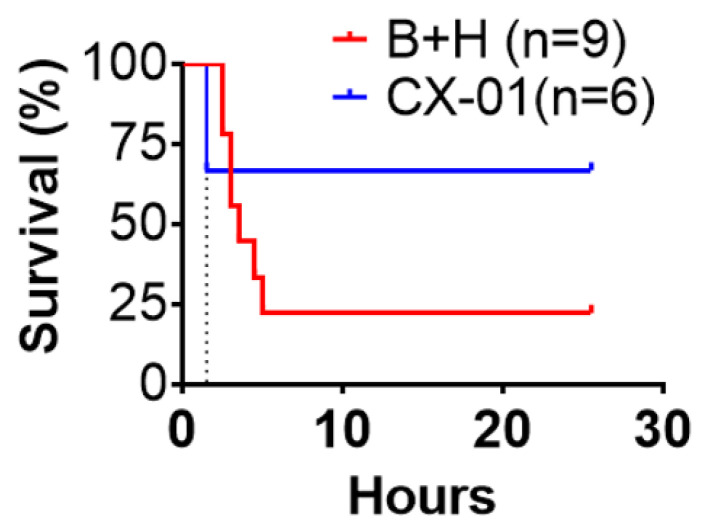
Effect of CX-01 treatment on injury survival in the rat model. Animal survival was monitored up to 24 h after TH. Survival distribution of these two groups was assessed by the log-rank Mantel-Cox test (*p* = 0.24).

**Figure 8:**
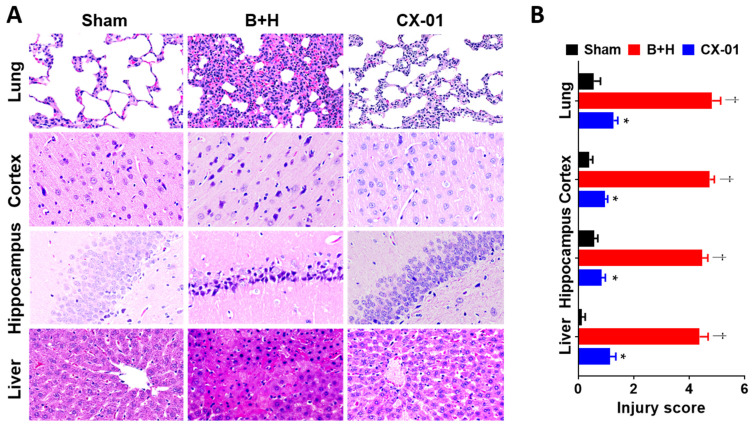
Effect of CX-01 treatment on histological changes in rats after TH. (A, B): Representative H&E photomicrographs of organs harvested at the time of necropsy (**A**) and organ injury were scored based on the criteria described in the Section 2 (**B**). The data are presented as mean ± SEM; * = *p* < 0.05 CX-01 vs. B + H; † = *p* < 0.05, Control vs. B + H (using the Mann–Whitney U test).

Treatment with CX-01 tended to improve survival (Figure 7). Histological assessment revealed that CX-01 treatment reduced MOF severity following TH. After TH, extensive cellular inflammatory infiltrates and obvious alveolar septal thickening were present in the pulmonary tissue; the majority of these were neutrophils and macrophages. Lung edema was severe after TH but CX-01 treatment alleviated this phenomenon (Figure 8A). Typical neuronal apoptosis, neuronal loss, and neuronal degeneration with morphological features consisting of shrinkage of cell body, pyknosis of nucleus, disappearance of nucleolus and loss of Nissl substance, were seen in cerebral cortex and hippocampus, which were significantly improved by CX-01 treatment (Figure 8A). TH induced hepatic tissue damage characterized by hepatic cell apoptosis, degeneration and necrosis. CX-01 treatment alleviated hepatic tissue damage (Figure 8A). Semi-quantitative scoring of injury severity on histology clearly demonstrated significantly lower injury score in CX-01-treated group as compared to BI + H group (*p <* 0.05) and further validated these observations (Figure 8B).

## 4. Discussion

In this study, we used a translational medicine approach to identify HMGB1, a damage-associated molecular pattern (DAMP), as a potential therapeutic target in a cohort of military casualties. We selected a heparin derivative (CX-01) that blocks HMGB1 activity and that has properties suitable for prehospital use in trauma patients. Finally, we assessed the efficacy of CX-01 on morbidity and mortality in rats subjected to blast injury and hemorrhagic shock.

We first investigated the relationship between HMGB1 blood concentrations, inflammatory mediators, and clinical outcomes in military casualties. We found significantly increased plasma concentrations of HMGB1 in the casualties on arrival to the hospital. Since field care and transportation of the casualties to the hospital took only 15–45 min in our study, this suggests that injury causes prompt release of HMGB1. In some reports, eHMGB1 has been considered a relatively delayed mediator of inflammation when compared with TNF and IL-1 [29]. On the other hand, HMGB1 concentration was increased in the serum 1 h after bilateral femur fracture in mice [7]. Similarly, in patients with mechanical trauma (ISS ≥ 15), plasma HMGB1 was increased more than 30-fold when compared to healthy controls within 1 h of injury; marked elevations were observed from 2 to 6 h after trauma. In contrast to late involvement of HMGB1 in endotoxin lethality in mice [46], it was released early after blunt or penetrating trauma [47]. In severe trauma patients without previous coagulation abnormalities, Cohen et al. showed that plasma levels of HMGB1 increased within 30 min after severe blunt or penetrating injury [48]. In a recent study in severe trauma patients, Ottestad et al. showed a biphasic release of HMGB1; a second peak 3–6 h after trauma was the most reliable predictor of outcome [49].

What is the cause and what is the effect of early eHMGB1 release following injury? HMGB1, a nuclear protein, has multiple functions that depend on its location in nucleus, cytosol, or extracellular space. Movement of HMGB1 between these compartments is a dynamic process, and it can be actively or passively released into the extracellular space during trauma, hemorrhagic shock, stress, and/or sepsis [20,22,50]. eHMGB1 occurs release after sepsis and trauma-induced inflammatory responses (cytokine storm, neutrophil extracellular trap formation, etc.), delivery of LPS into the cytoplasm, pyroptosis, oxidative stress, platelet activation and thrombosis, and endothelial/mucosal barrier dysfunction [20]. The recognition of eHMGB1 by pattern-recognition receptors (PRRs), such as the cell-surface receptor for advanced glycation end products (RAGE), and toll-like receptors (TLR2 and TLR4) promotes inflammation. eHMGB1 causes inflammation by itself or in complex with other pro-inflammatory molecules (e.g., DNA, RNA, histones, nucleosomes, lipopolysaccharide, SDF-1 IL-1α, IL-1β) [51]. When HMGB1 acts alone as a pro-inflammatory cytokine, the redox status of HMGB1 has a crucial role [51]. Mild HMGB1 oxidation, generating a disulfide bond between Cys23 and Cys45 while maintaining Cys106 in the reduced form, transforms eHMGB1 into a powerful catalyst of the generation of pro-inflammatory cytokines via TLR4 receptor stimulation [52].

HMGB1 can be a harmful self-derived molecule, the major DAMP that is recognized by PRRs secreted by immune cells or expressed on them [53,54,55]. Thus, HMGB1 triggers a sterile inflammatory reaction which leads to priming and activation of so-called NOD-, LRR-, and pyrin domain-containing protein 3 (NLRP3) inflammasomes, and, in turn, NLRP3-inflammasome activation results in active HMGB1 release (55). This inflammation pathway is activated via the axis of HMGB1-TLR2/4/RAGE-NF-κB-NLRP3/caspase-1, and/or HMGB1-CXCL12-CXCR4-NF-κB-NLRP3/caspase-1 [26,27,55,56,57,58].

Crosstalk between eHMGB1 and complement cascade has been suggested. Growing evidence in IRI indicates that their interaction is found through the axis of eHMGB1-properdin (a positive regulator of complement alternative pathway) [59], C5a-C5aR2-NF-κB-NLRP3/caspase-1-HMGB1 [60], or C5a-C5aR2-MAPK-HMGB1-TLRs/RAGE-MAPK-NLRP3/caspase-1 [61]. The interaction between HMGB1 and complement cascade may play an important role in TH-induced inflammatory response and inflammation-mediated MOF after TH. The relationship between eHMGB1 and the complement system has also been examined in IRI studies. These studies have reported that HMGB1-C1q interaction initiates complement activation and regulates inflammatory responses [62,63]; the complement receptor C5aR2 has been shown to contribute to NLRP3 inflammasome activation and HMGB1 release from murine macrophages in vitro and in vivo [60,61].

Consistent with the crosstalk concept, in military casualties, we showed that elevated HMGB1 levels were associated with complement alternative (Bb)/terminal pathway activation (C5a), MPO plasma levels, and plasma levels of a pro-inflammatory chemokine (MCP-1) and pro-inflammatory cytokines (IL-6, IL-8), as well as anti-inflammatory cytokines (IL-10, IL-13). Early plasma elevations of HMGB1 were also associated with some clinical outcomes, including GCS, ISS, SIRS, blood base deficit, coagulopathy, and fresh frozen plasma and fluid resuscitation requirements.

In injured rats, we observed that the CH50 value was reduced about 70% one hour after hemorrhagic shock. This decrease in CH50 indicates rapid systemic complement activation after injury. Using the same trauma model, we have previously showed complement deposition in the lung tissue, including C5b-9 and C3 (unpublished data). These findings indicate that both systemic and local complement activation occurred in injured animals. A clearly increased CH50 (up to 150%) at the end of the observation period coincided with inflammation in target tissues, and a significant increase in organ-injury scores. Gruys et al. explained highly increased CH50 as an indicator of the systemic acute-phase immune response that involves increased pro-inflammatory cytokines and C3 [64].

In our study, we found that CX-01, an inhibitor of HMGB1 that blocks systemic HMGB1 release/activity, reduced early complement consumption and therefore, complement activation; CX-01 also reduced consumption of C1q but there were no effects on C3 levels. A molecular mechanism of CX-01 in the inhibition of complement activation and C1q consumption in this study might be due to the potentiation of C1 inhibitor activity by CX-01 [65].

In this study, we also observed very high plasma HMGB1 levels at 4 h post-TH, indicating that blast/hemorrhage induces massive tissue/organ damage in rats. Our finding of the flat HMGB1 curve at the basal level in the CX-01-treated injured animals was unexpected. A plausible explanation is that CX-01-HMGB1 binding in vivo may prevent HMGB1-specific antibodies in the HMGB1 ELISA to detect HMGB1 in the samples. This postulated CX-01-HMGB1 binding is not only a phenomenon that would affect the HMGB1 measurement, but also even more importantly would block HMGB1 from activating its proinflammatory PRRs (RAGE, TLR2, TLR4, etc.) on macrophages/monocytes [36,37,38,39].

Previous studies have demonstrated that early administration of CX-01 increased survival and ameliorated lung injury in mice after *Pseudomonas* infection [41]. It also reduced brain damage and neuroinflammation, and improved acute neurologic recovery in mice after TBI [39]. Aligned with these findings, in this study we demonstrated that early administration of CX-01 improves survival and significantly attenuates TH-induced histological damage in lung, brain, and liver. CX-01, one of the minimal anticoagulant desulfated heparins, does not cause heparin-induced thrombocytopenia but retains multiple anti-inflammatory properties, including inhibition of RAGE-HMGB1 interaction [66]. Exploratory in vitro studies in the context of tumor immunotherapy have shown that CX-01 may also interfere with the CXCL12/CXCR4 axis [67]. There are other approaches to HMGB1 activity inhibition, including anti-HMGB1 antibodies. These studies have reported that inhibition of the HMGB1-mediated inflammatory signals improved outcomes in TBI and hemorrhagic shock [68,69].

Taken together, our clinical and preclinical findings show that HMGB1 plays a critical role in the pathogenesis of trauma and hemorrhage, and that CX-01 may be a promising pharmacological solution for the treatment of severely injured patients in pre-hospital settings. Although treatment with CX-01 clearly reduced tissue damage in some target organs of the injured rats, verifying the effect of CX-01 on survival would require a larger number of rats. Likewise, confirmation of such an effect would require studies in large animals.

## Figures and Tables

**Table 1:** Blast wave parameters. Legend: B + H group = blast + hemorrhage; CX-01 group = blast + CX-01; P0 (peak pressure) in kPa (the kilopascal, a unit of pressure); t+ (the positive-pressure phase duration in milliseconds (ms)); I (impulse (kPa-ms)).

	Reference			Overpressure			Reflected		
	P0 (kPa)	t+ (ms)	I (kPa-ms)	P0 (kPa)	t+ (ms)	I (kPa-ms)	P0 (kPa)	t+ (ms)	I (kPa-ms)
B+H (n = 9)	109.38 ± 1.27	3.31 ± 0.01	140.26 ± 0.86	117.26 ± 1.36	3.29 ± 0.01	141.38 ± 0.87	161.40 ± 2.07	3.48 ± 0.03	178.46 ± 1.35
CX-01 (n = 6)	109.58 ± 1.70	3.40 ± 0.03	143.92 ± 0.38	117.48 ± 1.82	3.38 ± 0.03	145.07 ± 0.39	162.00 ± 2.07	3.58 ± 0.01	182.15 ± 0.87

## Data Availability

All data generated or analyzed during the current study are included in this published manuscript.
